# Identification of Multiple Noise Sources Improves Estimation of Neural Responses across Stimulus Conditions

**DOI:** 10.1523/ENEURO.0191-21.2021

**Published:** 2021-07-03

**Authors:** Alison I. Weber, Eric Shea-Brown, Fred Rieke

**Affiliations:** 1 Graduate Program in Neuroscience, University of Washington, Seattle, WA 98195; 2 Department of Applied Mathematics, University of Washington, Seattle, WA 98195; 3 Department of Physiology and Biophysics, University of Washington, Seattle, WA 98195

## Abstract

Most models of neural responses are constructed to reproduce the average response to inputs but lack the flexibility to capture observed variability in responses. The origins and structure of this variability have significant implications for how information is encoded and processed in the nervous system, both by limiting information that can be conveyed and by determining processing strategies that are favorable for minimizing its negative effects. Here, we present a new modeling framework that incorporates multiple sources of noise to better capture observed features of neural response variability across stimulus conditions. We apply this model to retinal ganglion cells at two different ambient light levels and demonstrate that it captures the full distribution of responses. Further, the model reveals light level-dependent changes that could not be seen with previous models, showing both large changes in rectification of nonlinear circuit elements and systematic differences in the contributions of different noise sources under different conditions.

## Significance Statement

Current models for neural responses typically focus on accurately estimating a neuron’s average response to a stimulus but often fail to accurately reflect response variability. Such variability is central to the accuracy with which neural responses represent inputs and with which they can guide behavior. We present a new modeling framework that accurately captures observed variability in neural responses and find that multiple stochastic model elements are necessary to capture this variability. We show that model parameters can be accurately estimated using ∼8 min of data. We then apply the model to retinal ganglion cells, demonstrating light level-dependent changes in both deterministic and stochastic model elements changes that are either obscured or absent using more standard modeling approaches.

## Introduction

Variability in neural responses can reveal aspects of circuit function that are not apparent from average responses alone. For example, identifying different sources of variability can guide the search into potential mechanisms that shape neural responses (Softky and Koch, 1993; Franks et al., 2003; Goris et al., 2014). Variability also places fundamental limits on the information that can be encoded in single neurons (Faisal et al., 2005) and populations (Abbott and Dayan, 1999; Averbeck et al., 2006), and limits the accuracy of perception and behavior (Barlow, 1956; Bays, 2015). Finally, variability shapes how information flows through neural circuits, including the strategies used by neural circuits to mitigate the effects of noise (van Rossum and Smith, 1998; Butts and Goldman, 2006; Brinkman et al., 2016). To advance our understanding of circuit function, it is therefore important to develop models that provide more accurate predictions of variability in neural responses and better reflect the multiple origins of variability in neural circuits.

In order to accurately reflect neural variability, models must capture features that drive neural responses, as well as noise in the underlying mechanisms that produces true randomness. Ultimately, all these elements will be necessary to produce more complete models that disentangle the contributions of different mechanisms to neural responses. To date, much work has focused on incorporating into models additional stimulus features that drive spiking, dependence on response history, or modulation from other neurons (Berry and Meister, 1998; Keat et al., 2001; Slee et al., 2005; Fairhall et al., 2006; Pillow et al., 2008). Comparatively little work has focused on modeling the noise inherent in neural responses (Keat et al., 2001; Churchland et al., 2011; Vidne et al., 2012; Goris et al., 2014).

In current models, the generation of spikes from a neuron’s inputs is most commonly described as a Poisson process (Dayan and Abbott, 2001; Schwartz et al., 2006), potentially with a refractory period or otherwise modulated by response history (Berry and Meister, 1998; Pillow et al., 2008). However, the assumption that variability in neural responses takes the form of Poisson noise arising in spike generation has several limitations. First, it is inconsistent with the fact that variability in neural circuits arises from multiple sources at different stages of processing (Faisal et al., 2008). Second, Poisson noise is of a particular magnitude: the variance of responses is equal to the mean. Yet variability can differ in magnitude depending on stimulus conditions and the neural circuit in question, ranging from sub-Poisson (Berry and Meister, 1997) to strongly super-Poisson (Shadlen and Newsome, 1998). The typical assumption of Poisson noise can lead to systematic biases in the estimation of underlying circuit computations, such as receptive fields and nonlinearities (Pillow and Simoncelli, 2003). These shortcomings suggest the need for new models that incorporate diverse stochastic elements and can flexibly adapt to match the strength and statistics of noise observed under different stimulus conditions or in different systems. Beyond simply reproducing observed responses with greater accuracy, such models guide the search for circuit mechanisms driving observed variability (Churchland et al., 2011; Goris et al., 2014).

Here, we take the approach of incorporating stochastic model elements inspired by what is known about the biological circuitry and likely sources of noise. We present a model that includes multiple potential sources of noise, which may arise at different locations relative to a circuit nonlinearity and which may have distinct effects on the observed variability in responses. This model allows us to estimate the strengths of these individual noise sources and clarify the separate contributions of deterministic and stochastic model elements. We first demonstrate that this model can be tractably fit to a dataset of limited size and that we accurately recover model elements, both the shape of the nonlinearity and the strength of each noise source, in a simulated dataset with known model parameters. We then demonstrate an application of this model in retinal ganglion cells (RGCs). The model captures response variability under two different stimulus conditions and further reveals consistent changes in both the nonlinearities and inferred sources of noise that depend on stimulus condition. The model is suitable for a variety of systems and allows for comparisons across stimulus conditions, revealing changes that are either obscured or absent using more standard modeling approaches.

## Materials and Methods

### Experimental procedures

All animal procedures were performed in accordance with the Institutional Animal Care and Use Committee at the University of Washington. Experiments were performed on whole mount preparations of retina from overnight dark-adapted C57/BL6 mice (ages 5–20 weeks). All procedures were conducted under infrared illumination to preserve dark adaptation. Retinas were mounted ganglion cell-side up onto a poly-D-lysine-coated coverslip (BD Biosciences) before being placed in a recording dish that was continuously perfused at 7–9 ml/min with oxygenated Ames bicarbonate solution (Sigma) warmed to 31–34^∘^C. Spike responses were recorded from RGCs using extracellular or loose-patch recordings with an Ames-filled pipette. Visual stimuli were presented on an OLED microdisplay monitor (eMagin) focused onto the photoreceptors. Stimuli were presented and data acquired using custom-written stimulation and acquisition software packages Stage (http://stage-vss.github.io) and Symphony (http://symphony-das.github.io). On-sustained and Off-sustained RGCs were targeted for recording based on their large soma size (>20 μm in diameter) and responses to light increments and decrements. All recordings were from cells that responded reliably with more than five spikes to 100-μm diameter spots presented for 500 ms at 20% contrast on a background of 10 R*/rod/s. For both mean light levels (10 and 1000 R*/rod/s), Gaussian noise stimuli were presented as spatially uniform spots 200 μm in diameter at 50% contrast. The contrast of the spot was changed every 67 ms (four frames at a monitor refresh rate of 60 Hz). Noise stimuli that were modulated at higher temporal frequency did not robustly drive cells at 10 R*/rod/s. Cells were adapted to each new light level for at least 8 min and until responses to flashed spots stabilized before recording. For On-sustained cells, five cells were recorded at both light levels, three cells at only low light, and one cell at only high light. For Off-sustained cells, two cells were recorded at both light levels.

### Data analysis

Linear filters were found by standard reverse-correlation methods: calculating the spike-triggered average and correcting for autocorrelation in the stimulus. Filters were smoothed by low-pass filtering with a frequency cutoff of 13 Hz. For each cell, identical filters were used for the model with Poisson noise [linear-nonlinear-Poisson (LNP) model] and the multistage noise model. For cells in which data were collected at two different light levels, separate filters were calculated at each light level, with filters at higher light levels being faster and more biphasic than those at low light, consistent with previous work (Enroth-Cugell and Lennie, 1975). Throughout this work, filtered stimulus values are z-scored to make comparisons across cells and conditions.

Both the filtered stimulus and responses were divided into time windows of ∼60–100 ms, in which the average filtered stimulus was taken as the input to the model and the spike count was taken as the response. The exact length of the time window for a cell at a given light level was determined by the shape of the linear filter and corresponded to twice the width of the filter at half-max. This duration was chosen to produce minimal correlation between filtered stimulus values in neighboring bins. Bins of this length also minimize spike history effects because of refractoriness, which are expected on shorter timescales.

Models with Poisson noise (LNP models) are given by:

(1) $$r_{t}=\text{Pois}\left(f\left(x_{t}\right)\right),$$

where *r_t_* is the neuron’s response (spike count) in time bin *t*, *x_t_* is the average filtered stimulus value in time bin *t*, *f* is the nonlinearity, and $\text{Pois}(f\left(x_{t}\right))$ is a Poisson random variable with mean $f\left(x_{t}\right)$. The nonlinearity is parameterized as a softplus function:

(2) $$f(x)=\beta_{1}\ln\left(1+e^{\beta_{2}x+\beta_{3}}\right)+\beta_{4}.$$

This is done for consistency with the multistage noise model presented below, in which the nonlinearity is parameterized this way. This function was chosen because it can capture the range of desired features in a nonlinearity, from highly rectified to effectively linear. We see little or no evidence of response saturation at high input values in our data and therefore did not choose a sigmoidal (saturating) nonlinearity. This parameterization does not reduce the model’s ability to capture mean responses: on repeated stimulus presentations, the correlation coefficients between actual mean responses and predicted responses were 0.98 and 0.87 for the example cells shown at low and high light, respectively, regardless of whether the nonlinearity was a parameterized softplus function or was instead a nonparametric function found by locally estimated scatterplot smoothing (LOESS), a method of locally weighted regression. Model parameters for LNP models are found by maximum likelihood estimation, using the same routine described below for the multistage noise model.

### Multistage noise model

**Figure 1.**
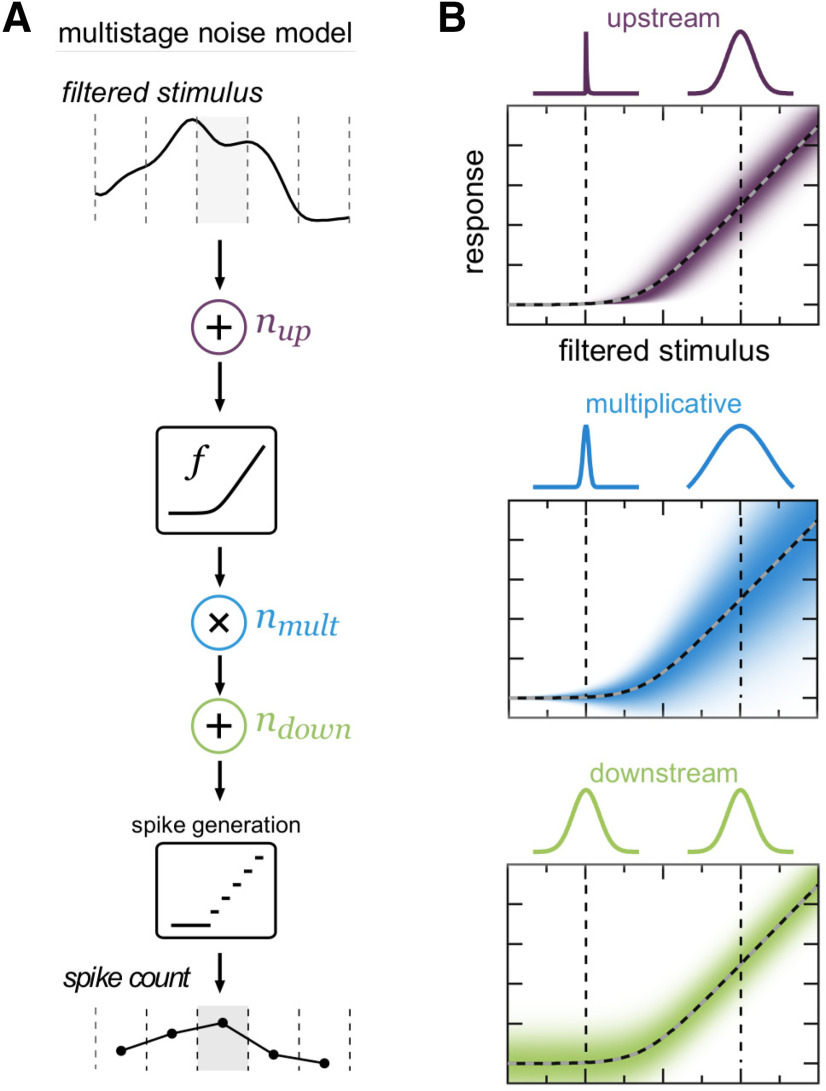
Multistage noise model. ***A***, A linearly filtered stimulus is corrupted by additive noise, passed through a softplus nonlinearity that experiences multiplicative noise, and then corrupted by additional additive noise. Spikes are generated by finding the nearest nonnegative spike count. ***B***, Illustration of the effects of potential sources of noise before spike generation. Color intensity indicates relative probability of responses. Colored lines above each plot indicate conditional response distribution for filtered stimulus value indicated by corresponding vertical dashed line. Top, Gaussian additive noise upstream of the nonlinearity is smeared out by the nonlinearity (dashed gray and black line), resulting in greater noise in the responses for areas of greater sensitivity (higher slope) in the nonlinearity. Middle, Gaussian multiplicative noise at the output of the nonlinearity scales with the output of the nonlinearity. Bottom, Additive noise downstream of the nonlinearity produces distributions that are independent of the nonlinearity input and output.

The model we present here (Fig. 1*A*) incorporates multiple sources of variability to reflect the varied sources of noise present in neural circuits:

(3) $$r_{t}=\text{R}\left[n_{mult,t}\cdot f\left(x_{t}+n_{up,t}\right)+n_{down,t}\right].$$

*r_t_* is the spike count in time bin *t*, and *x_t_* is the average filtered stimulus in time bin *t*. R rounds and rectifies to produce a spike count. The nonlinearity *f* is a softplus function, parameterized as in Equation 2. There are three noise sources: two additive and one multiplicative (Fig. 1*B*). The two additive noise sources are termed “upstream” and “downstream” noise to indicate their positions relative to the nonlinearity. For simplicity, noise sources are generally taken to be Gaussian (with an exception noted in the following section):

(4) $$n_{up,t}∼N\left(0,\sigma_{up}^{2}\right)\;n_{mult,t}∼N\left(1,\frac{\sigma_{mult}^{2}}{f\left(x_{t}+n_{up,t}\right)}\right)\;n_{down,t}∼N\left(0,\sigma_{down}^{2}\right).$$

For noise that fluctuates on fast timescales relative to data binning, noise is expected to follow Gaussian distributions because of the central limit theorem. When all sources of noise are Gaussian, the model has seven total parameters: four that determine the shape of the nonlinearity and three that determine the strength of the noise sources.

### Modifications to the multistage noise model

**Figure 2.**
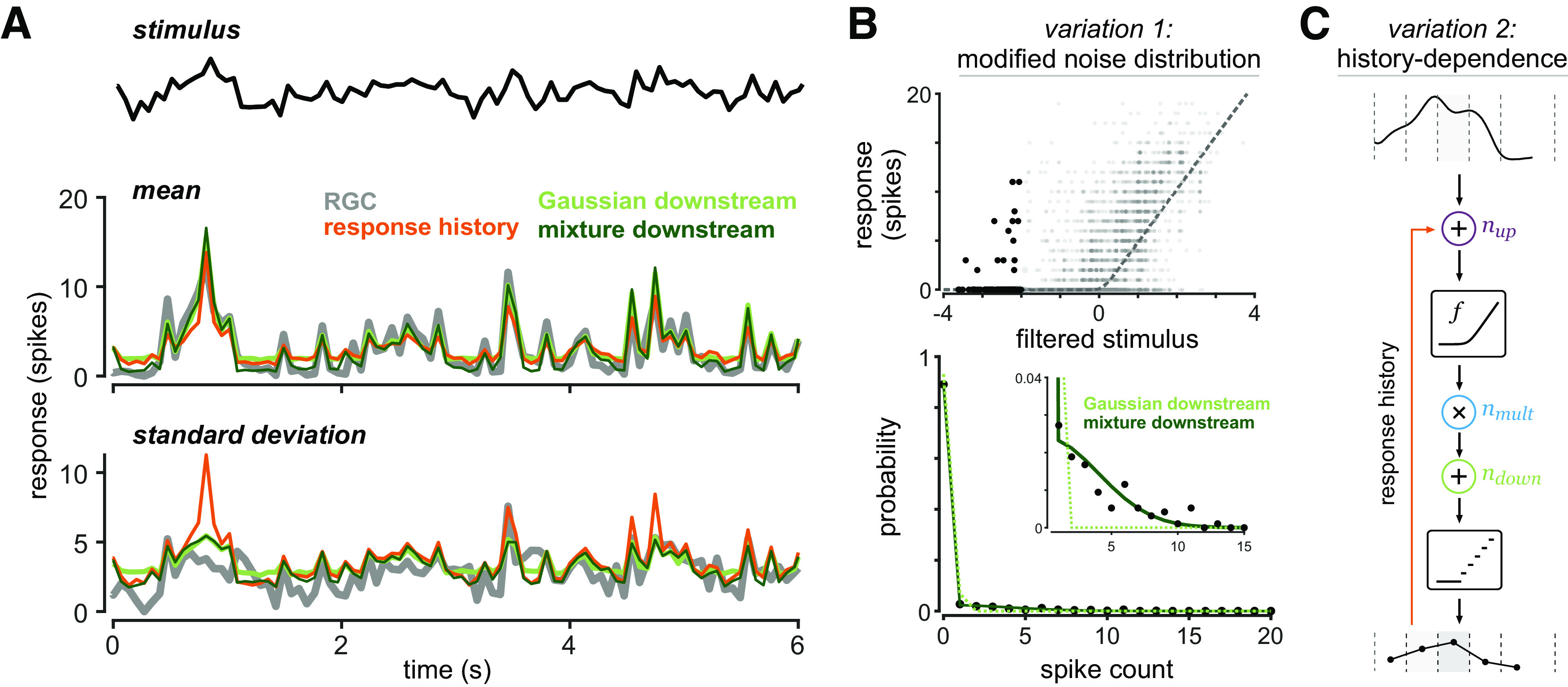
Modifications to the multistage noise model. ***A***, RGC average response and response SD on repeated trials, with predictions from three model variants: Gaussian downstream noise, Gaussian downstream noise with response history, and Gaussian mixture downstream noise (without spike history). ***B***, Model variation with data-driven modification of the downstream noise distribution. Model architecture as in Figure 1*A*. Top, Data for a single cell at high light (same as Fig. 3*B*), with points corresponding to input values less than 2 SDs below the mean in black. Bottom, Distribution of points highlighted in top panel, with best-fit rectified Gaussian and rectified Gaussian mixture distributions. ***C***, Model variation that incorporates response history dependence. Input to the nonlinearity is given by the filtered stimulus value plus the weighted spike response in the previous time bin plus the upstream noise.

**Figure 3.**
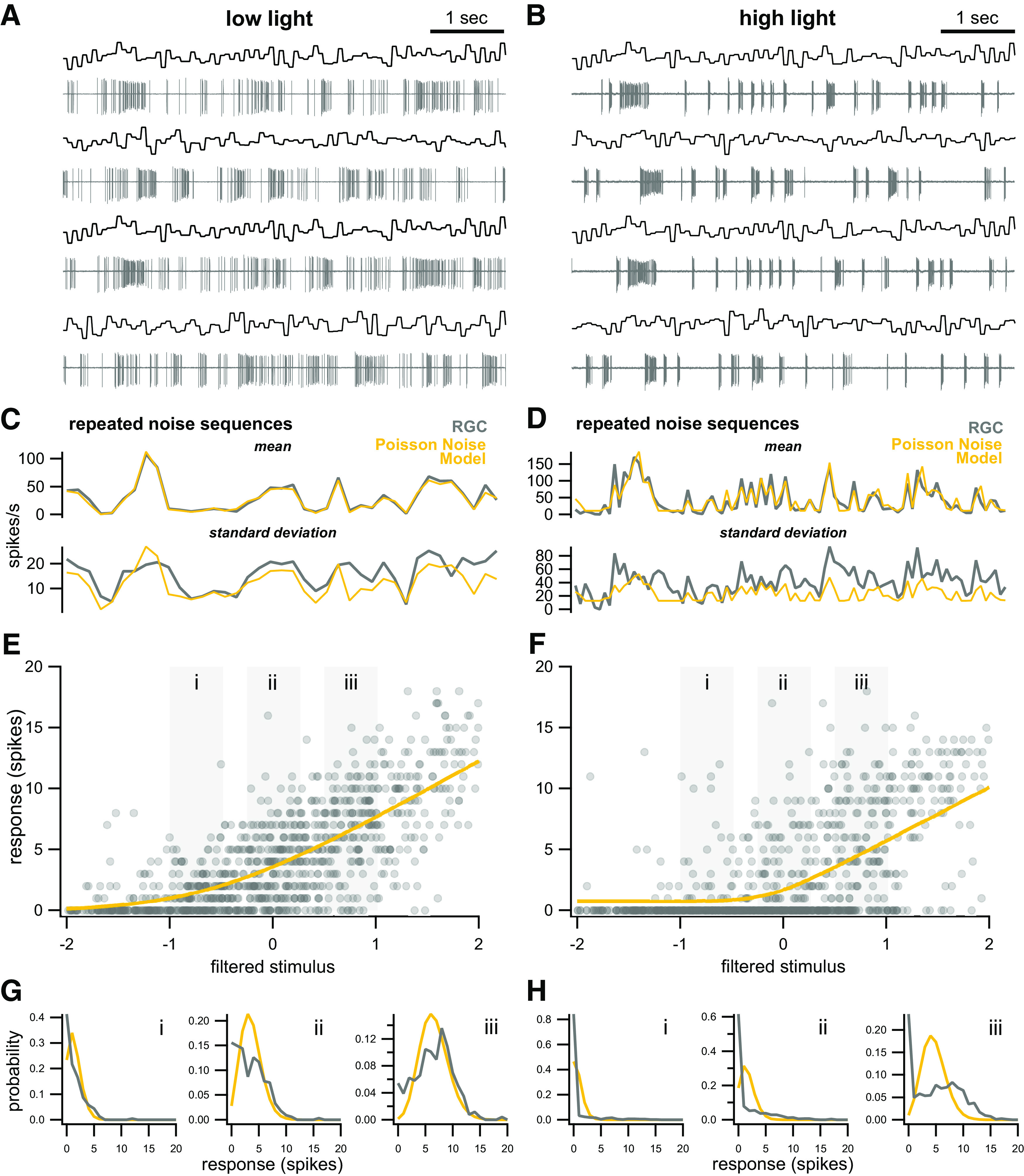
Recordings from RGCs at two different light levels, with predictions from models with Poisson noise. ***A***, ***B***, Example responses of On-sustained RGCs to presentation of alternating repeated and random noise sequences; 50% contrast, mean light level 10 R*/rod/s and 1000 R*/rod/s, ***A***, ***B***, respectively. ***C***, ***D***, RGC responses to repeated presentations of the same noise sequence (gray) and best-fit linear-nonlinear model with Poisson noise (yellow). Top, Mean measured responses and predictions. Bottom, SD of measured responses and predictions. ***E***, ***F***, Neural responses to non-repeated noise sequences plotted as a function of filtered stimulus values. Yellow line indicates best-fit nonlinearity for a linear-nonlinear model with Poisson noise. ***G***, ***H***, Distributions of neural responses (gray) from corresponding gray boxes in ***E***, ***F***. Yellow distributions are those predicted by the LNP model.

Under some stimulus conditions (high light level, in particular), the multistage noise model with all Gaussian sources of noise did not accurately capture responses (Fig. 2*A*). Such a model will not be able to capture response distributions like those in Figure 2*B*, bottom panel, with predominantly zero responses but a long tail of nonzero responses. We therefore investigated variations of the model that might improve predictions. Given that the linear-nonlinear framework accurately captures average responses on repeated trials (Fig. 2*A*; also Fig. 3*C*,*D*), we did not expect that the model would be improved by additional deterministic elements that affect stimulus selectivity. Adding additional stimulus features that drive spiking has proven useful in some contexts, but will not improve the model’s ability to capture the observed response variability.

We therefore turned our attention to model elements that could alter the model’s predicted variability. We tested whether our multistage noise model might be improved by adding response history dependence. In this model variant, input to the nonlinearity was given by the sum of the filtered stimulus value, an upstream noise value, and the spiking response in the previous time bin weighted by a factor determined by the optimization (Fig. 2*C*), resulting in one additional parameter. This slightly improved the model prediction of average responses, but resulted in higher predicted variation than that seen in the data (Fig. 2*A*). As is the expectation for a Poisson generalized linear model (GLM), our multistage noise model attributes a positive weight to the response history to capture super-Poisson variability, attributing some amount of variability to this process. Notably, this is in contrast to what spike history effects are typically thought to achieve, namely reduced variability via refractory effects (Berry and Meister, 1998; Keat et al., 2001).

Because the model predictions are not improved by incorporating response history dependence, we next considered changing the distributions of the noise sources directly. We used observed features of the responses to guide our choice of a new noise distribution. Specifically, at low input values, where the nonlinearity is flat and produces an output near zero, nearly all noise is expected to be contributed by the downstream noise source. Whereas response distributions for these low input values were approximately rectified Gaussian distributions at low light (corresponding to purely Gaussian downstream noise rectified by spike generation), response distributions at high light were well described by a mixture distribution:

(5) $$n_{down,t}=\{\begin{array}{ll} N∼N\left(0,\sigma_{down}^{2}\right) & \text{with probability }p_{down}\\ 0 & \text{with probability }1-p_{down} \end{array}.$$

This distribution can account for the large number of zero responses present at low input values (Fig. 2*B*). The mixture distribution can be thought of as representing an intermittent source of noise: some portion of the time (given by *p_down_*) this source of noise is present, while the remainder of the time it is absent. This might reflect the fact that this source of noise is itself engaged by a noisy process that takes effect randomly throughout stimulus presentation. Note that the original model, with purely Gaussian downstream noise, is a subset of this model. This modification adds one additional parameter to the model, for a total of eight parameters.

### Estimating model parameters from data

In brief, a combination of C++ and MATLAB code was used to find the maximum likelihood estimate of model parameters. What follows is the likelihood function for this model, broken down to reflect each step in the model for clarity. The full likelihood function can be found by plugging functions from preceding steps into Equation 9.

Let *P_up_*, *P_mult_*, and *P_down_* denote the probability distributions of each noise source. To calculate the likelihood of an observed spike count *r_t_*, first the distribution reflecting the input plus upstream noise is passed through the nonlinearity *f*. *P_up_* is shifted by (or equivalently centered on) the observed input *x_t_*. The distribution of outputs from the nonlinearity *λ* is:

(6) $$P_{\Lambda }(\lambda )=P_{up}\left(f^{-1}(\lambda )-x_{t}\right)\cdot \frac{df^{-1}(\lambda )}{d\lambda }.$$

The distribution *P*_Λ_ is a distorted version of *P_up_* that spreads out where the derivative of the nonlinearity is large (derivative of the inverse is small) and compresses where the derivative of the nonlinearity is small (derivative of the inverse is large). See Miller and Childers (2012) for a more thorough treatment of transformations of probability distributions. Note that this equation holds for monotonically increasing nonlinearities.

After multiplicative noise is applied, the distribution is given by the following:

(7) $$P_{Y}(y)=\int_{-\infty }^{\infty }P_{\Lambda }(\lambda )\cdot P_{mult}(y-\lambda )d\lambda .$$

This is similar to a convolution, except that the standard deviation of *P_mult_* increases with *λ*. This has the effect of spreading the distribution more for larger values of *λ* where the multiplicative noise distribution is wider.

The distribution after downstream noise is given by the following:

(8) $$P_{Z}(z)=\int_{-\infty }^{\infty }P_{Y}(y)\cdot P_{down}(z-y)dy.$$

This reflects a simple convolution of *P_Y_*with the downstream noise distribution.

Finally, we integrate to obtain the probability of observing integer spike counts:

(9) $$P_{R}(x_{t},r_{t})=\{\begin{array}{ll} \int_{-\infty }^{0.5}P_{Z}(z)dz & r_{t}=0\\ \int_{r_{t}-0.5}^{r_{t}+0.5}P_{Z}(z)dz & r_{t}>0 \end{array}.$$

We treat each observed response *r_t_* as independent of responses at other times. The full likelihood L is therefore simply the product of *P_R_*(*x_t_,r_t_*) (the probability of an individual observation at time *t*) over all time points:

(10) $$L=\prod_{t}P_{R}(x_{t},r_{t}).$$

Note that the total recording time most strongly determines the number of data points available for fitting (along with the time window chosen for binning), rather than the number of spikes recorded, as is often the case for other models.

The maximum likelihood estimate of parameters was found using the Nelder–Mead method (implemented by MATLAB’s fminsearch function). This provided the best performance among several optimization methods tested. A small modification to the fminsearch function allowed for bounds on parameters (fminsearchbnd, John D’Errico). Bounds were used only to ensure that impossible regions of parameter space (e.g., negative SDs for noise sources) were not explored, rather than to constrain the optimization to a subset of desired parameter values. As this problem is not guaranteed to have a unique solution, for each dataset we began the optimization from 5 to 10 different randomized initial conditions. For noise parameters, initial conditions were drawn randomly from a uniform distribution of possible values. For nonlinearity parameters, parameters found by least-squares fitting were perturbed randomly by ±40% to set initial conditions. This amount of perturbation allowed for variability while ensuring that the initial conditions produced a plausible shape for the nonlinearity. The solution with the highest likelihood was reported. Although it is not necessary to perform this procedure to estimate parameters of the model with Poisson noise, the same procedure was used to make a fair comparison with the multistage noise model.

Several steps were taken to speed evaluation of the likelihood function. Equation 7 was evaluated at individual points using custom C++ code that makes use of the quadratic adaptive integration package (integration_qag) of the GNU Scientific library (https://www.gnu.org/software/gsl/). The full function of Equation 7 was approximated with Chebyshev polynomials using the Chebfun package for MATLAB (Driscoll et al., 2014; http://www.chebfun.org/). Machines running the Ubuntu operating system with multiple cores (16 or 40) were used to run the optimization in parallel with the Parallel Computing Toolbox in MATLAB.

### Code accessibility

The code described in the paper is freely available online at https://github.com/aiweber/Multistage_noise_model. The code is also available as Extended Data 1.

10.1523/ENEURO.0191-21.2021.ed1 Extended Data 1 Code that fits the multistage noise model to data. See README file for additional details. Download Extended Data 1, ZIP file.

### Calculation of signal-to-noise ratio (SNR)

In several figures, we indicate the level of each noise parameter that corresponds to an SNR of 0.5 to provide intuition for the strength of each noise source. Because the contribution of a single noise parameter to the overall SNR will depend on both the strength of other noise sources as well as the shape of the nonlinearity, here we calculate SNR for each noise source individually (i.e., with other noise sources set to zero) and using either the true nonlinearity (in the case of simulated data) or the estimated nonlinearity (in the case of retinal data).

We calculate the SNR as follows:

(11) $$\text{SNR}=\frac{{\text{Var}}_{s}\left[E[r|s]\right]}{E_{s}\left[\text{Var}[r|s]\right]},$$

where the innermost expectation (numerator) and variance (denominator) are taken over all responses *r*, conditioned on the stimulus *s*. The outer variance (numerator) and expectation (denominator) are then taken over the stimulus distribution.

### Jensen–Shannon divergence (JSD)

The JSD is a measure of similarity of probability distributions (Lin, 1991), which we use to assess how closely response distributions produced by our model match experimentally observed response distributions. It is a symmetric modification of the Kullback–Leibler divergence and guaranteed to have finite value for all probability distributions.

(12) $$JSD(P,Q)=\frac{1}{2}\left[D_{KL}(P,R)+D_{KL}(Q,R)\right],$$

where $R=\frac{1}{2}\left(P+Q\right)$ and *D_KL_* is the Kullback–Leibler divergence:

(13) $$D_{KL}(Q,R)=-\sum_{x}Q(x)\log\left(\frac{R(x)}{Q(x)}\right)$$

## Results

Neural responses in many systems, including the retina, show non-Poisson variability and large changes in variability between stimulus conditions. Our goal is to understand the origins of this observed variability and how the contributions of different sources of variability change across conditions. Previous models typically capture mean responses well but do not accurately reflect response variability. We propose that variability can be better described by a model that incorporates multiple stochastic elements that represent plausible sources of noise in the biological circuit. Further, the relative contributions of these sources of noise suggest possible mechanisms giving rise to observed variability. We show that such a model can be tractably fit to data and then use the recorded responses of a well-studied neural population, retinal ganglion cells, as a benchmark for evaluating the model.

Before proceeding, it is important to clarify the distinction between variability and noise. Noise refers to inconsistency in responses that arises because of stochastic processes and is considered to obscure the signal of interest. Noise is therefore generally (although not always) unfavorable from the perspective of neural coding. Noise can be considered a subset of variability, which more broadly refers to some inconsistency in a neuron’s response and hence could include uncontrolled experimental variables, such as behavioral state or temperature. In the context of neural recordings, we refer to stochastic biological processes as producing noise but generally discuss variability in neural responses, as the sources of the variation are not fully known at the level of neural outputs. Variability in model responses arises entirely from stochastic model components, and hence we refer to it as noise.

### Observed variability in neural responses deviates from Poisson and depends on light level

We observed that responses of RGCs exhibit variability that consistently deviates from Poisson variability and depends on light level. We first recorded responses from On-sustained RGCs of the mouse at two different levels of ambient illumination while presenting spatially uniform Gaussian noise stimuli (200-μm diameter spot, 50% Weber contrast, centered on the soma; Fig. 3*A*,*B*). On-sustained ganglion cells were chosen because they are easily identified by their large soma size and characteristic responses to light increments. We were therefore able to target a single cell type with a high degree of accuracy. At the lower level of illumination (10 R*/rod/s) responses are primarily rod-mediated, while at the higher level of illumination (1000 R*/rod/s) responses are primarily mediated by cones (Dedek et al., 2008; Cowan et al., 2016).

Cells were presented with alternating repeated and random noise sequences. Average responses to repeated presentations of the same noise sequence are shown in Figure 3*C*,*D*. These responses serve as an important benchmark for evaluating the accuracy of a model, as they provide direct estimates of average responses and response variability that do not rely on any model assumptions.

We then characterized the ganglion cell responses as a function of linearly filtered stimulus values, a common simplification that often captures response selectivity in ganglion cells well (Chichilnisky, 2001; Kim and Rieke, 2001). We first used standard reverse-correlation methods to compute the linear filter that best relates the stimulus to the observed responses. Applying this filter to the stimulus yields the best linear prediction of responses, often called the “generator signal.” Figure 3*E*,*F* show RGC responses in a short time window (∼100 ms) plotted against the average filtered stimulus in the same time window at low and high light, respectively. In both cases, it is apparent that the average response increases as a function of the filtered stimulus values, although there is a great deal of variability in responses to a given input.

We next found the nonlinear function that best predicts the neural response (spikes in a small time window) as a function of the filtered stimulus. We parameterized the nonlinearity as a softplus function; this choice does not reduce the accuracy with which responses are predicted relative to a smoothed, nonparametric estimate of the nonlinearity (Materials and Methods). We first made use of the most common assumption for neural response variability: that spike responses follow Poisson distributions. Figure 3*C*,*D* show that while this LNP model produces accurate estimates of the average responses on repeated trials (*R* = 0.98 and *R* = 0.87 for low and high light, respectively), it does not accurately capture the response variability across trials, particularly at high light. Figure 3*G*,*H* similarly show that the response distributions over a small range of filtered stimulus values are not well-described by Poisson statistics, but rather show super-Poisson variability. Deviations from Poisson statistics have been well-documented in a number of previous studies (Berry and Meister, 1998; Pillow et al., 2005), although retinal responses generally show sub-Poisson rather than super-Poisson variability.

Not only do response distributions show deviations from Poisson predictions at both light levels, but the deviations are qualitatively different in each of these cases. Comparing across the two light levels, it is apparent that the distribution of responses for a given filtered stimulus value is very different, even when the mean output of the nonlinearity is similar. Compare, for example, Figure 3*Giii* and *Hiii*, where the predicted output of the nonlinearity is similar (approximately five spikes). In particular, there is a far greater probability of observing zero spikes at high light compared with low light. Such a difference reflects underlying changes in how the circuit is operating in these two conditions, changes that may not be apparent by focusing only on mean responses and that cannot be accounted for by any model in which spikes are taken to follow Poisson statistics. In these models, the response distribution is entirely determined by the mean, and hence any two stimuli that produce the same mean responses would produce identical response distributions. A model that could provide insight into changes in circuit function under these conditions thus ought to have the flexibility to capture different distributions of responses for identical input values.

### Model of variable neural responses with multistage noise

These observations indicate that variability in neural responses is driven by mechanisms that cannot be accurately modeled by simple Poisson noise. To help identify which mechanisms might account for this variability, we sought a model that would more accurately reflect the potential sources of variability at multiple circuit locations to account for observed response distributions. Because the linear-nonlinear framework accurately captures average responses to repeated white noise sequences, we built on these deterministic elements to allow for differences in response variability.

One likely candidate for model improvement is dependence on response history. If response history is able to modify input to the nonlinearity, it can effectively alter the level of variability in responses such that a model with Poisson noise and response history dependence, such as Poisson GLMs, may exhibit sub-Poisson or super-Poisson variability in its outputs (Berry and Meister, 1998; Keat et al., 2001; Weber and Pillow, 2017). However, even if a Poisson GLM were able to accurately capture response distributions, it would do so in a way that does not reflect known sources of noise in biological circuits: by positing Poisson noise at the output step and attributing all deviations from Poisson variability to the effects of spike history. Much of the variability in the retinal output signals, however, is inherited from sources in upstream circuits (Murphy and Rieke, 2008; Ala-Laurila et al., 2011; Freed and Liang, 2014). Moreover, GLMs encounter a practical problem when fitting datasets with high variability, as we see here at high light. Super-Poisson variability is achieved by positive spike history terms, where a spike at some previous time increases the probability of a spike at the current time. In practice, this can lead to runaway firing in the model (Weber and Pillow, 2017).

Predicted response variability can also be altered by incorporating stochastic elements other than noise in spike generation. We sought to modify the linear-nonlinear model framework in this way, incorporating noise at different locations within the model. These stochastic model elements more accurately reflect that noise arises in several elements of neural circuits, rather than simply spike generation (Faisal et al., 2008). In fact, spike generation in the retina and elsewhere in the nervous system is near-deterministic (Bryant and Segundo, 1976; Mainen and Sejnowski, 1995; Murphy and Rieke, 2006). In the retina specifically, noise is known to arise at several distinct stages of processing, including within the photoreceptors (Schneeweis and Schnapf, 2000; Ala-Laurila et al., 2011; Angueyra and Rieke, 2013; Field et al., 2019) and at the bipolar cell output synapses (Freed, 2000; Dunn and Rieke, 2006; Borghuis et al., 2009; Freed and Liang, 2014).

In our new model, we incorporated three potential sources of noise into a linear-nonlinear cascade framework to reflect the varied sources of noise present in neural circuits (Fig. 1*A*). Noise arising at different circuit locations, before and after the nonlinearity, will have distinct effects, even if both sources of noise are additive and drawn from the same distribution. The sources of noise in the model are intended to capture different features of experimentally observed variability (stimulus-dependence or -independence, additive or multiplicative effects) while remaining tractable to fit to data. Changes in the relative magnitude of these different noise sources can give rise to models that produce different response distributions even when the mean output is identical.

In this multistage noise model (Fig. 1*A*), the filtered stimulus first encounters additive noise, which we refer to as upstream noise (*n_up_*) to indicate its position relative to the nonlinearity in the model. After the corrupted input is passed through the nonlinearity, it encounters multiplicative noise (*n_mult_*), in which the output of the nonlinearity is multiplied by a random noise value. This is followed by another source of additive noise downstream of the nonlinearity (*n_down_*). The output of this step is a continuous, unbounded prediction of the response in the time window of interest. Rather than introducing an additional noisy spike generation step, we take the nearest nonnegative integer as the predicted spike count. This deterministic method of spike generation reflects the fact that spike generation itself accounts for little of the variability observed in neural responses (Bryant and Segundo, 1976; Mainen and Sejnowski, 1995; Murphy and Rieke, 2006). Note that this model allows us to estimate both the shape of the nonlinearity and the strengths of each noise source (unlike, for example, LNP models or GLMs, in which the noise component of the model is fixed).

In some cases, the model nonlinearity will be strongly determined by a single nonlinearity in the biological circuit. In the retina, for example, rectification at the bipolar to ganglion cell synapse dominates the nonlinearity observed in the ganglion cell’s responses (Schwartz and Rieke, 2011; Grimes et al., 2014). However, the nonlinearity does not necessarily reflect a particular biophysical feature of the circuit (e.g., rectification at a particular synapse or an individual cell’s spike threshold). It nevertheless provides a useful description of the circuit’s selectivity to the preferred stimulus feature: stronger nonlinearities and steeper slopes are indicative of greater selectivity to the feature given by the linear filter. Providing an accurate estimate of this nonlinearity is therefore informative of circuit function, even when it does not correspond to a particular location in the circuit.

The effects of each noise source on response variability are illustrated in Figure 1*B*. Each panel shows the distribution of model outputs (shown before spike generation for clarity) when only a single source of noise is present, with subpanels above illustrating the conditional distributions at filtered stimulus values marked by dashed vertical lines. For simplicity, all sources of noise depicted are Gaussian, although outputs of the model are not Gaussian-distributed because of effects of the nonlinearity and spike generation.

Although the magnitude of upstream noise is independent of the input, its effects are magnified by regions of high sensitivity (high slope) in the nonlinearity and eliminated by flat regions (far left). The effects of multiplicative noise scale with the output of the nonlinearity. Because the noise is multiplied by the output of the nonlinearity, larger output values result in greater variability. This is similar to Poisson noise, where output variance equals the mean output, although this multiplicative source of noise has greater flexibility in that output variance scales with the mean by a constant factor (not constrained to be 1). Both upstream and multiplicative noise therefore result in variability that depends on the input: response variability due to upstream noise scales with the derivative of the nonlinearity, and response variability due to multiplicative noise scales with the output value of the nonlinearity itself. Downstream noise is independent of the input and thus results in equally variable responses across all regions of the nonlinearity. In summary, the three sources of noise produce different signatures of variability in the responses that can be uniquely identified.

### Demonstration of the effects of each source of noise in the model

To demonstrate the effects of each noise source in the multistage noise model, we simulated data from three models with identical deterministic properties but each with one of the non-Poisson noise sources described above. For comparison, we then fit a model that assumes Poisson noise to demonstrate the errors produced by a mismatch between the actual and assumed location of noise. Systematic errors are expected in this case, but we demonstrate the specific issues that arise for the three different types of noise in the multistage noise model to provide intuition for the results that follow.

**Figure 4.**
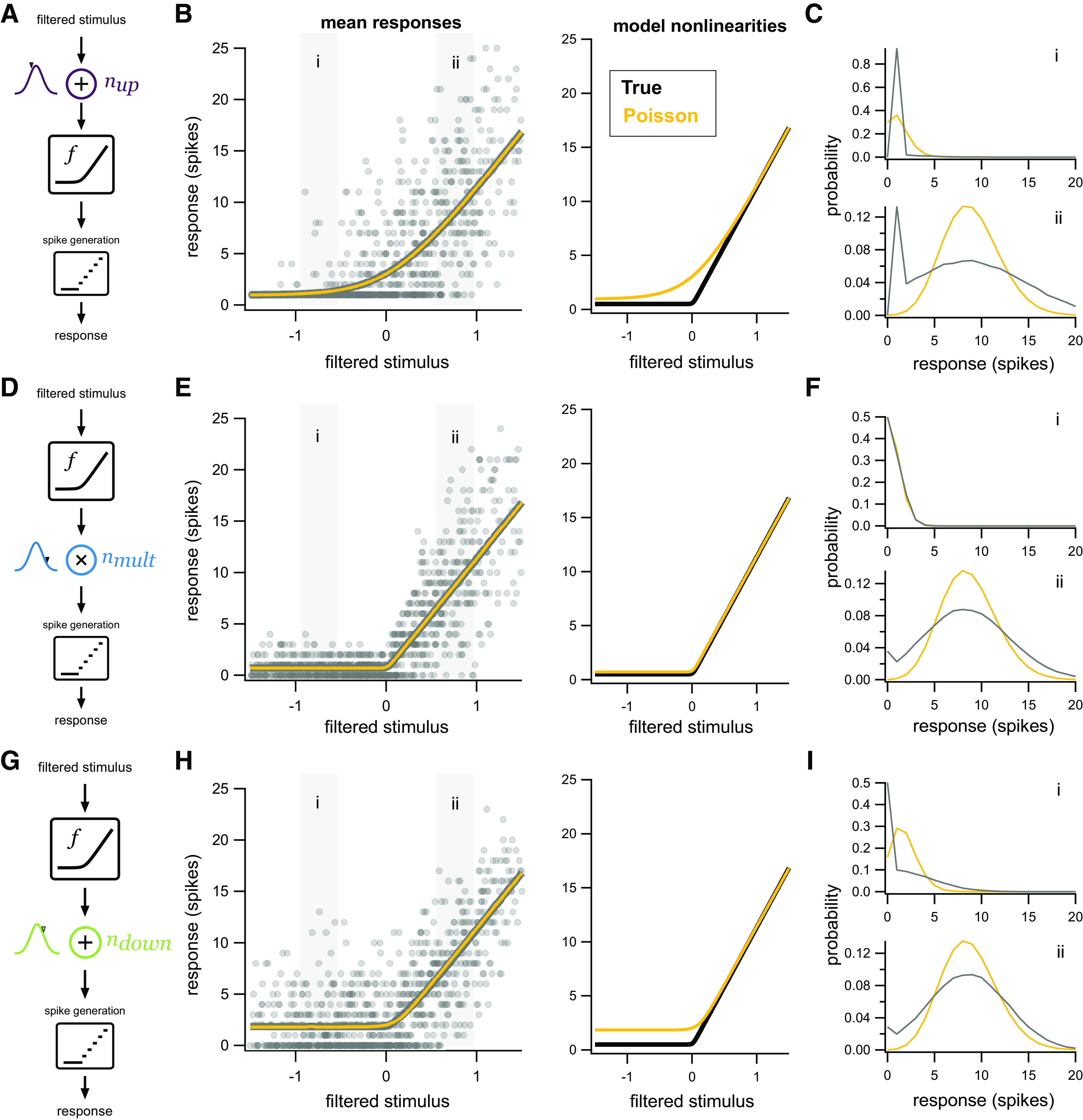
Summary of the effects of different sources of noise. ***A***, Model schematic as in Figure 1, except with only a single source of noise: Gaussian additive noise upstream of the nonlinearity. ***B***, Left, Simulated data (gray dots) with only additive Gaussian upstream noise. The mean responses predicted by a linear-nonlinear model with Poisson noise (yellow) closely track the mean responses in the simulated data (gray). Right, The nonlinearity inferred by the LNP model (yellow) is systematically biased from the nonlinearity used to generate the data (black). ***C***, Distributions of responses (gray) from corresponding gray boxes in ***B*** compared with those predicted by the LNP model (yellow). ***D–F***, Same as ***A–C***, except with only multiplicative Gaussian noise at the output of the nonlinearity. ***G–I***, Same as ***A–C***, except with only additive Gaussian noise downstream of the nonlinearity.

If only additive Gaussian noise upstream of the nonlinearity is present and an LNP model is fit to the resulting responses (Fig. 4*A–C*), the inferred nonlinearity will be less sharply rectified (i.e., more linear) than the true underlying nonlinearity that produced the data. Noise added to a filtered stimulus will make responses to that input, on average, more similar to nearby inputs, having the effect of “smearing” out the nonlinearity. Response distributions will be poorly fit across all filtered stimulus values.

Note that for an LNP model, the nonlinearity in the model is identical to the mean predicted response, so the yellow lines in the left and right panels of Figure 4*B* are identical. For other models, such as a model with additive noise upstream of the nonlinearity, the true model nonlinearity (black, right panel) does not necessarily trace out the mean responses (gray, left panel) predicted by the model.

If only multiplicative Gaussian noise is present, the inferred nonlinearities and response distributions may be well approximated by Poisson noise (Fig. 4*D–F*). In both cases, the variance of responses scales with the output. In the example depicted, the multiplicative noise scales with 1.5 times the output of the nonlinearity and is thus slightly super-Poisson, hence the clear discrepancy in Figure 4*Fii*. The nonlinearity has a small vertical offset such that even negative input values produce a small positive nonlinearity output. This small positive output then interacts with multiplicative noise to create variability in the observed spike counts at these low input values.

If only additive Gaussian downstream noise is present (Fig. 4*G–I*), the inferred nonlinearity will exhibit a prominent vertical offset at low input (filtered stimulus) values. At higher input values, responses are Gaussian-distributed and Poisson noise approaches Gaussianity, so there is little effect on the estimated nonlinearity. (If downstream noise were non-Gaussian, however, there would be a greater discrepancy between response distributions.) At low input values, mean responses fall well above zero even when the true nonlinearity is zero. This occurs because downstream noise is rectified and hence produces nonnegative spike counts and a vertical offset of the estimated nonlinearity.

These examples demonstrate the impact that assumptions about noise can have on the inferred shape of the nonlinearity: incorrect assumptions can lead to strongly biased estimates of the nonlinearity operating in the circuit.

### Estimating multistage noise model parameters

One key feature of the linear-nonlinear model with Poisson noise is its simplicity to fit to data, requiring only standard reverse correlation methods to find the linear filter and least-squares estimate of the nonlinearity (Chichilnisky, 2001). Given the relative complexity of our proposed multistage noise model, it is unclear whether it is tractable to fit to data or whether there is a unique set of parameters that best characterize a given dataset. To answer these questions, we generated simulated data from the multistage noise model with known parameters and then estimated parameters of the simulated data to determine whether they were accurately recovered. We generated simulated datasets of a size corresponding to only ∼8 min of data collection (5000 points, corresponding to roughly 8 min of data assuming a linear filter width of 100 ms), generally shorter than the recordings we have from RGCs to which we wish to fit the model.

We used a maximum likelihood approach to estimate model parameters. In order to reduce computation time, we first approximated the likelihood function and then used standard optimization methods to find the maximum of this function. Importantly, parameters for both the nonlinearity and noise are estimated simultaneously, as these interact to determine the likelihood. As demonstrated above, incorrect assumptions about the structure of noise in a circuit can bias estimates of the nonlinearity.

Because the likelihood function is non-convex, optimization is not guaranteed to arrive at the maximum likelihood set of parameters. We therefore begin our optimization at several different initial conditions (5–10) and select the parameters that result in the overall greatest likelihood (Materials and Methods). In practice, initial conditions often converge to similar parameter estimates, suggesting that the likelihood does not have many deep local minima. Using this procedure, we find that we are able to estimate model parameters with a high degree of accuracy.

**Figure 5.**
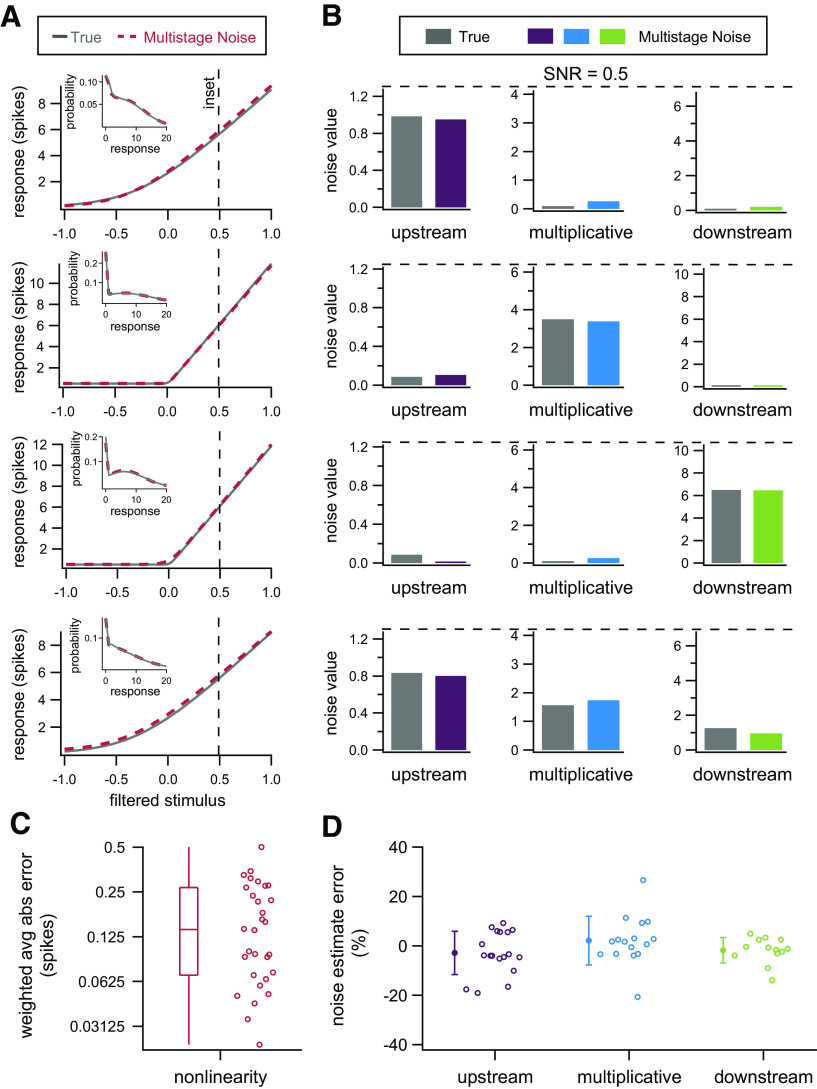
Model parameters can be accurately recovered from simulated data. ***A***, Nonlinearities used to generate four example datasets (gray) and nonlinearities recovered by fitting the multistage noise model (red). Insets, Distributions of responses at the input value indicated by the dashed vertical line for the dataset (gray) and multistage noise model (red). ***B***, True (gray) and recovered (purple, blue, green) parameters for the four example datasets. The upper limit on each vertical axis corresponds to an SNR of 0.5 when the respective noise source is the only one present. ***C***, Average absolute error of inferred nonlinearities, weighted by the input distribution, for all 30 simulated datasets. ***D***, Error in estimated noise parameters for simulated datasets. Points are shown for all cases in which the corresponding source of noise contributed at least 20% of the total noise. Error bars denote SD.

We begin with a model in which all sources of noise are Gaussian distributed. Recall that the model still produces highly non-Gaussian spike counts in this case. Figure 5*A*,*B* show four example datasets: one where each source of noise dominates (top three rows) and one where all sources of noise contribute (bottom row). We are able to recover the nonlinearity that generated the data with high precision, as well as the sources of noise present in the data. We can therefore reconstruct with high precision the full distribution of responses at any given input value (Fig. 5*A*, insets).

To test the generality of these conclusions, we generated 30 simulated datasets with varying parameters, including both steep and shallow nonlinearities and different combinations of dominant noise sources. In these datasets, we can recover the nonlinearity that produced the data with a high degree of accuracy (Fig. 5*C*). The error in the recovered nonlinearities for the multistage noise model is nearly always <0.3 spikes on average; that is, for a given set of parameters the absolute difference between the output of the true nonlinearity and the recovered nonlinearity is <0.3 spikes averaged across the range of possible inputs. Across all parameter sets, the mean error in the recovered nonlinearity is 0.17 spikes and is always <1% of the total range of outputs. By comparison, the average error in the recovered nonlinearity under the assumption of Poisson noise is 1.06 spikes. As expected, the error under the Poisson assumption is markedly greater than the multistage noise model because the data are generated from models with different noise structure than the model used for fitting. Nonetheless, the Poisson noise case provides a useful point of reference for the error we might expect if incorrect assumptions are made about noise in fitting experimental data.

In addition to recovering the nonlinearity, the multistage noise model estimates the strength of each noise source to within 20% of its true value for all noise sources that contribute meaningfully to the response (Fig. 5*D*). Noise sources that contributed <20% of the total noise were excluded. These values are often estimated with large error (when measured as percentages), but do not markedly impact overall response variability because they are small in absolute terms. In summary, for a range of parameter values with a modestly sized dataset, we can accurately recover both the nonlinearity and the sources of noise that produced the data.

### Application of multistage noise model to RGCs: low light

**Figure 6.**
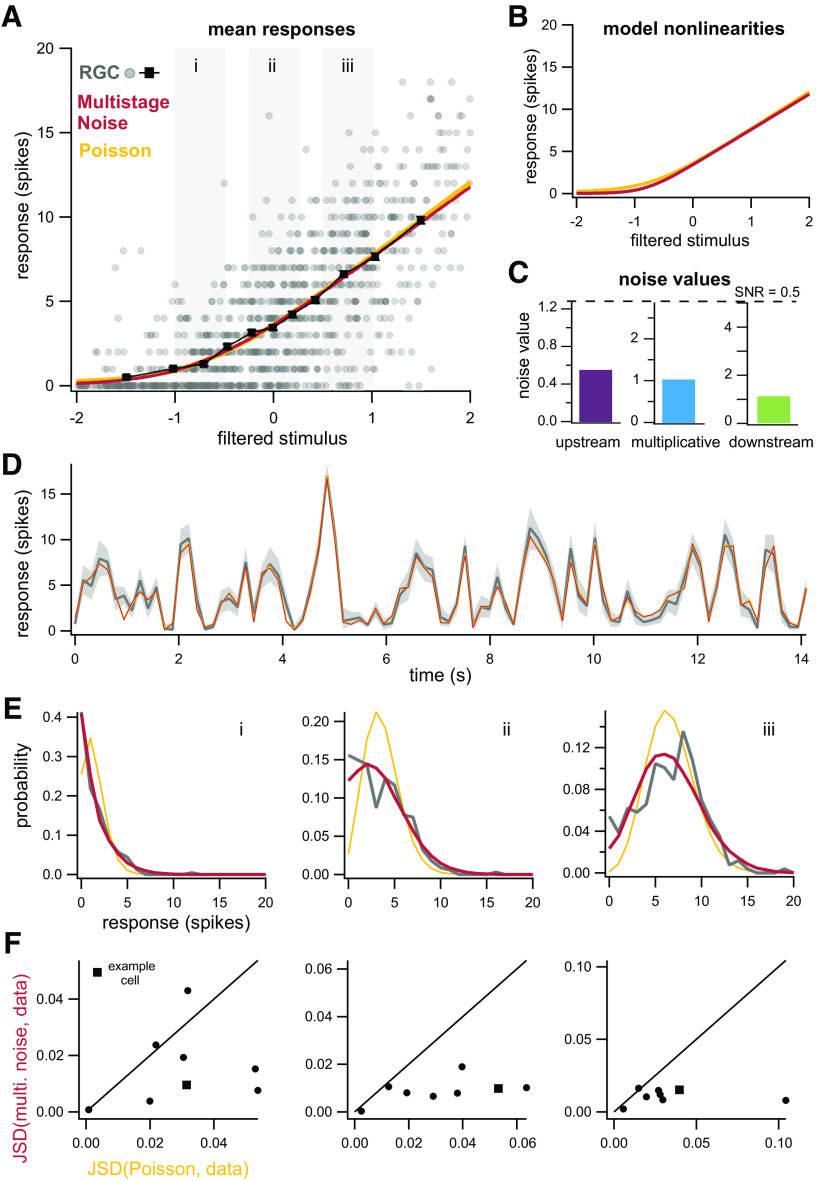
Multistage noise model accurately captures responses of RGCs at low light. ***A***, Mean responses predicted by both a linear-nonlinear model with Poisson noise (yellow) and multistage noise (red) are similar for this example cell and accurately predict the mean responses from the data (black). Error bars denote SEM. ***B***, Model nonlinearities for the LNP and multistage noise model are similar. ***C***, Noise values for each noise source in the multistage noise model. All noise sources contribute to observed variability. ***D***, Average responses of example cell from ***A*** for repeated trials of the same noise sequence (gray). Shaded area indicates bootstrapped 98% confidence intervals on the mean. Predictions of trial-averaged responses are similar for both models. ***E***, Distributions of responses from gray boxes in ***A***. ***F***, JSD between the distributions of responses from data and the multistage noise model, plotted against JSD between distributions from data and the LNP model, shown for eight different ganglion cells (circles, square for example cell in ***A–E***). Columns correspond to the ranges of filtered stimulus values indicated by gray boxes in ***A***.

We next fit the model to ganglion cell responses at low light levels (10 R*/rod/s) to determine whether it accurately captures observed response variability. For the example cell shown in Figure 6, the mean responses predicted by the multistage noise model are nearly identical to those predicted by a model with Poisson noise, shown for both for the full dataset as a function of filtered stimulus (Fig. 6*A*) and for responses to repeated presentations of the same noise sequence (Fig. 6*D*). Nonlinearities extracted by the two models are also nearly identical (Fig. 6*B*). Recall that for the multistage noise model, the model nonlinearity does not necessarily trace out the mean responses predicted by the model, although they are similar here.

When we consider the full distribution of responses, however, we see that the multistage noise model outperforms a model with Poisson noise. More specifically, a Poisson approximation is somewhat suitable at higher and lower filtered stimulus values (Fig. 6*Ei*,*Eiii*). The Poisson approximation fails more obviously near the center of the input distribution (Fig. 6*Eii*), where inputs are most probable (i.e., where most of the data lie).

We then applied this model to additional cells (*n* = 8), all exposed to the same level of ambient illumination (10 R*/rod/s). Results are summarized by plotting the JSD between the predicted and actual response distributions at three different input levels. JSD is a measure of difference between two probability distributions; lower JSD indicates better correspondence between two distributions. Across all filtered stimulus values, the JSD between the data and predictions from the multistage noise model is systematically lower than the data and the Poisson noise model (Fig. 6*F*).

### Estimating parameters for a variant of the model

The multistage noise model accurately captures average responses and response variability in the low light condition when all noise sources in the model are Gaussian. At high light, however, Gaussian noise sources were unable to account for the observed response distributions, particularly the long tail of nonzero responses at low input values (Figs. 2*B*, 3*Hi*). We found that modifying the noise distributions (specifically the downstream noise distribution), provided better fits to the response distributions than alterations of other model components (Fig. 2; Materials and Methods). In the modified model, downstream noise is Gaussian-distributed with probability *p_down_* and zero otherwise. This mixture distribution can be thought of as representing an intermittent source of noise that is present with probability *p_down_*. The original model, with purely Gaussian downstream noise, is a subset of this model.

**Figure 7.**
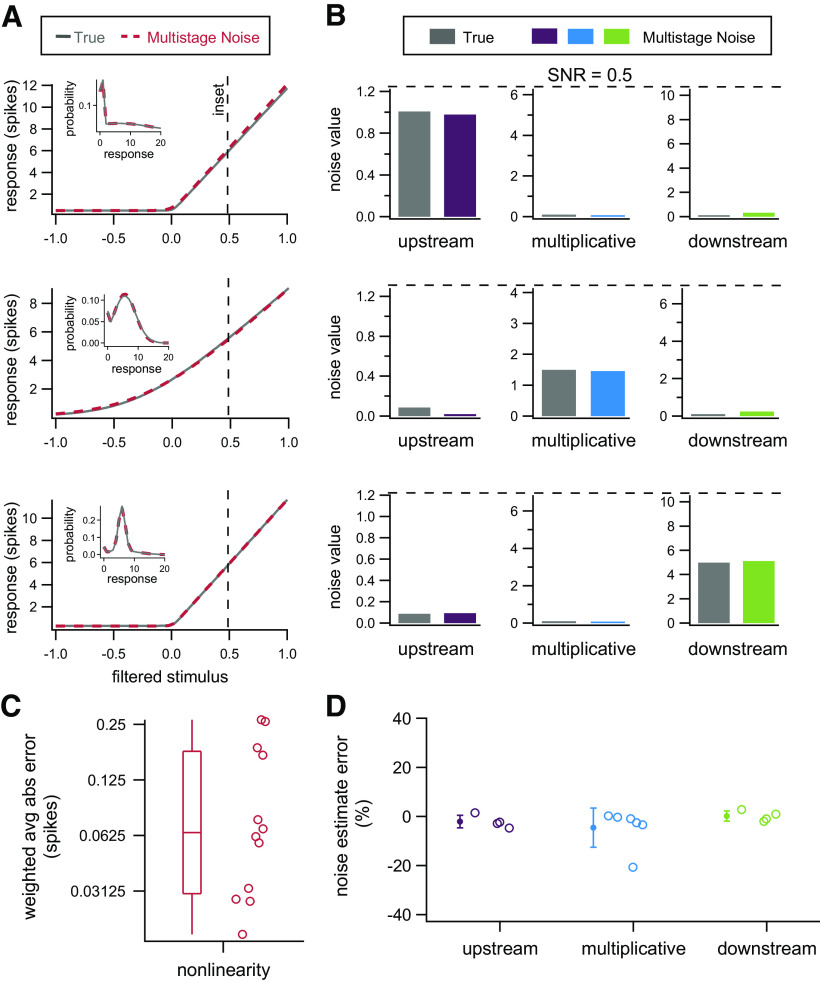
Model parameters can be accurately recovered from simulated data where downstream noise is drawn from a mixture distribution. ***A***, Nonlinearities used to generate example datasets (gray) and nonlinearities recovered by fitting the multistage noise model (red). Insets, Distributions of responses at the filtered stimulus value indicated by the dashed vertical line for the dataset (gray) and multistage noise model (red). ***B***, True (gray) and recovered (purple, blue, green) parameters for the three example datasets. ***C***, Average absolute error of inferred nonlinearities, weighted by the input distribution, for all 12 simulated datasets. ***D***, Error in estimated noise parameters for simulated datasets. Points are shown for all cases in which the corresponding source of noise contributed at least 20% of the total noise. Error bars denote SD.

In order to determine whether parameters of this model with modified downstream noise (which has one additional parameter) could also be recovered, we again generated simulated data from this model and used the same procedures to estimate model parameters. (The likelihood function is slightly altered because of the change in downstream noise distribution, but model fitting procedures are otherwise identical to the previous model.) Results for three example datasets and summary results across 12 simulated datasets are presented in Figure 7. As with the previous model, both nonlinearity and noise parameters can be recovered with high accuracy. For simplicity, we show the SD of the downstream noise distribution $\left(\sqrt{p_{down}\sigma_{down}^{2}}\right)$, but both parameters can be individually recovered with similar accuracy.

### Application of model to RGCs: high light

**Figure 8.**
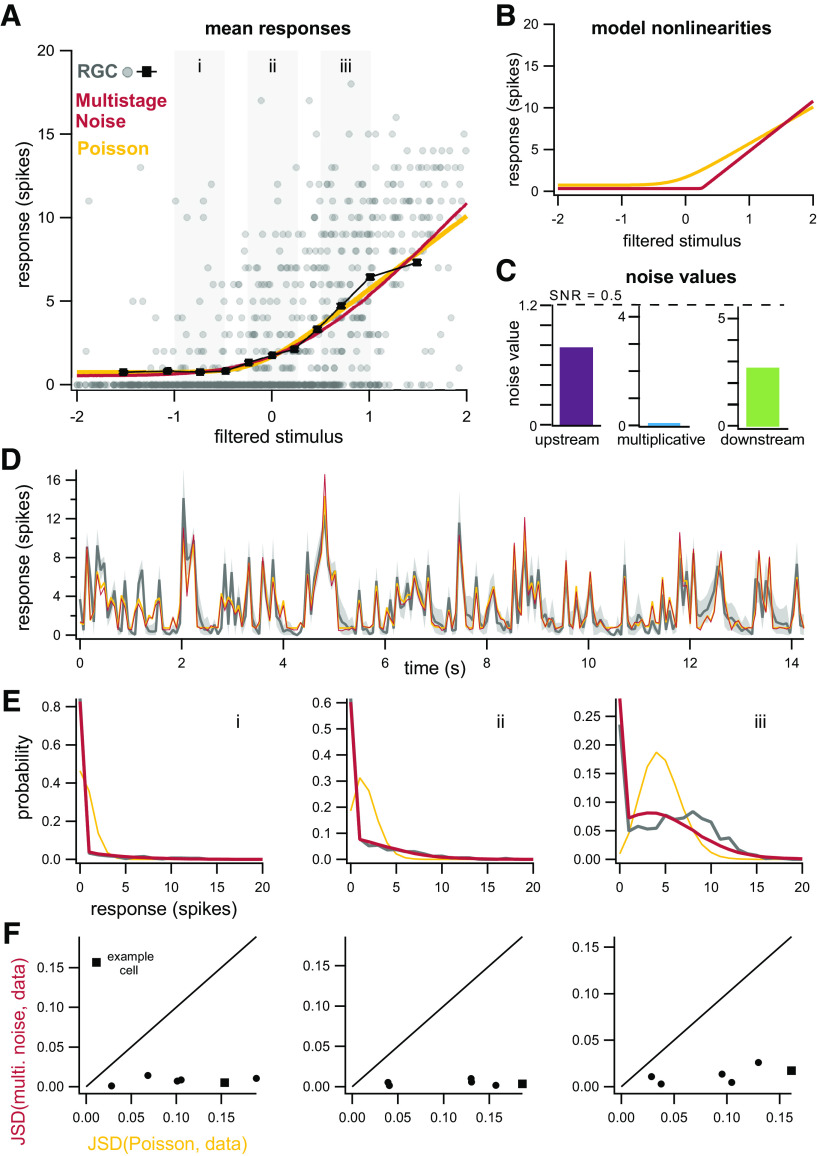
Model with downstream noise drawn from a mixture distribution accurately captures responses of RGCs at high light. ***A***, Mean responses predicted by a linear-nonlinear model with Poisson noise (yellow) and multistage noise (red) are again similar for this example cell and accurately predict the mean responses from the data (black). Error bars denote SEM. ***B***, Model nonlinearities are markedly different. ***C***, Upstream and downstream noise in the multistage noise model both contribute to the observed variability. ***D***, Average responses of example cell from ***A*** for repeated trials of the same noise sequence (gray). Shaded area indicates bootstrapped 98% confidence intervals on the mean. Predictions of trial-averaged responses are similar for the multistage noise model and LNP model. ***E***, Distributions of responses from gray boxes in ***A***. ***F***, JSD between the distributions of responses from data and the multistage noise model, plotted against JSD between distributions from data and the LNP model, for six different ganglion cells (circles, square for example cell in ***A–E***). Columns correspond to the ranges of filtered stimulus values indicated by gray boxes in ***A***.

We next fit the model to RGC responses at high light levels (1000 R*/rod/s). The nonlinearities inferred by the new model and a model with Poisson noise are markedly different, with the multistage noise model inferring a much more sharply rectified nonlinearity (Fig. 8*B*). Again, predictions of the average responses are nearly identical for both models (Fig. 8*A*,*D*), despite the differences in nonlinearities. Note that although the mean responses predicted by both models for large input values appear to fall below the cloud of points, these are actually accurate predictors of the mean responses because of the number of zero responses at large input values (Fig. 8*A*). For the cell shown, both upstream and downstream noise sources contribute to the observed variability (Fig. 8*C*).

The predicted distributions of responses for the multistage noise model are in close correspondence with the data, whereas Poisson distributions provide a poor approximation across input values (Fig. 8*E*). The multistage noise model is able to capture the large number of observed zero responses, unlike a model with Poisson noise. Model variants that incorporate additional stimulus features or response history, rather than multiple noise sources, are unable to account for the observed response distributions (Fig. 2; Materials and Methods). Across a population of cells (*n* = 6), the multistage noise model predicts the distribution of responses better than the Poisson noise model across all input levels (Fig. 8*F*).

### Comparison of model features at low and high light levels

We next sought to determine whether the model revealed systematic differences between ganglion cell responses, in either the nonlinearity or noise, when ambient illumination changes. Changes in these features may reflect changes in which circuit elements are engaged or adaptive changes in how particular circuit elements process inputs. In order to make fair comparisons between the two conditions, we fit data at both light levels using the multistage noise model with a mixture distribution for downstream noise.

**Figure 9.**
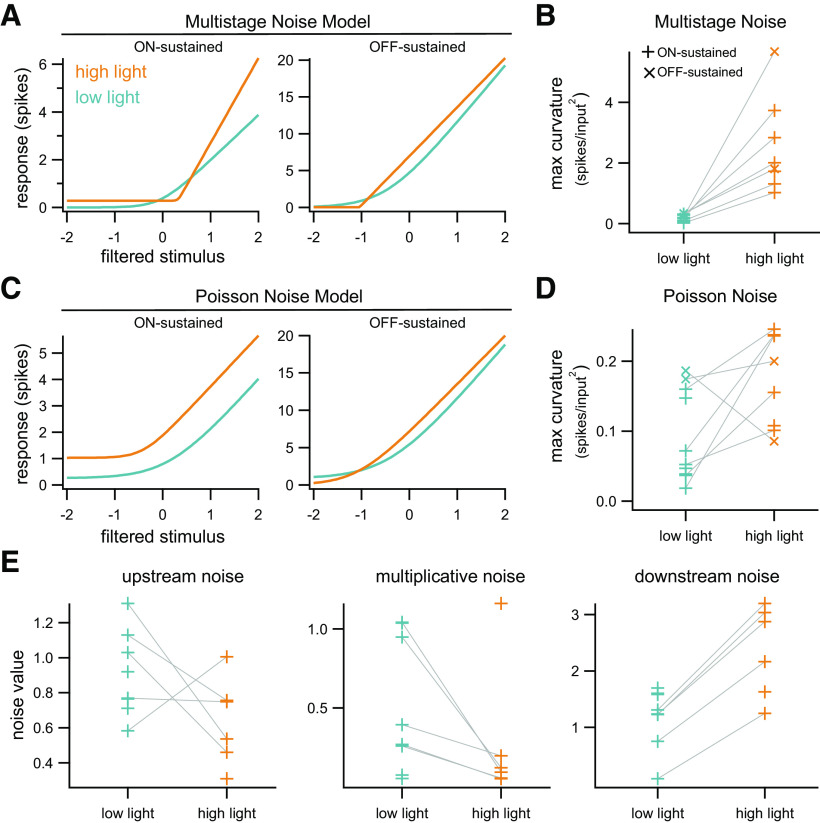
Comparison of model parameters at low and high light levels. ***A***, Nonlinearities at low (teal) and high (orange) light for one example On-sustained cell and one example Off-sustained cell, estimated with the multistage noise model. ***B***, Summary of maximum nonlinearity curvature, as measured by the second derivative, across the population (11 cells; +: 7 On-sustained; x: 2 Off-sustained) for the multistage noise model. Data points for individual cells that were recorded from at both light levels are connected by gray lines (7 cells). Nonlinearities at high light are consistently more sharply rectified than those at low light. ***C***, ***D***, Same as ***A***, ***B*** for model with Poisson noise. Note the different scale of the vertical axis in ***D*** compared with ***B***. ***E***, Inferred noise strengths at low and high light across the population of On-sustained cells. Individual cells show consistent differences across light levels for multiplicative and downstream noise.

For On-sustained RGCs, nonlinearities were consistently more sharply rectified at high light compared with low light, both for individual cells recorded at both light levels (Fig. 9*A*) and across the population of cells (average ratio high-to-low 12.95; *p* <0.001, Wilcoxon rank-sum; Fig. 9*B*). Curvature was quantified by taking the maximum of the second derivative of the nonlinearity. There is no possible scaling of the vertical or horizontal axis that overlays the nonlinearities in the two cases, ruling out the possibility that this change is simply because of differences in dynamic range or differences in the effective contrast experienced by the cell under these two conditions. In comparison, nonlinearities found assuming Poisson noise also show significantly stronger rectification at high light but are far more similar under the two conditions (ratio high-to-low 2.52; *p* = 0.01, Wilcoxon rank-sum; Fig. 9*C*,*D*). Further, these nonlinearities are far less sharply rectified than those found using the multistage noise model (compare scale of vertical axes in Fig. 9*B*,*D*). We also recorded two Off-sustained cells, which show similar trends to the On-sustained cells, with nonlinearities more strongly rectified at high compared with low light (Fig. 9*A*,*B*). These changes were not apparent in Off-sustained cells when assuming Poisson noise (Fig. 9*C*,*D*).

All three sources of noise present in the multistage noise model are needed to account for ganglion cell responses (Fig. 9*E*). There are no systematic differences in the magnitude of the upstream noise source across light levels (*n* = 5 cells with data at both light levels; average ratio low-to-high 1.56; *p* = 0.31, Wilcoxon signed-rank). Multiplicative noise, on the other hand, is lower at high light levels for all cells in which paired data are available (average ratio low-to-high 6.15; *p* = 0.06, Wilcoxon signed-rank). The inferred strength of the downstream noise source is higher at high light levels for all cells with paired data (average ratio low-to-high 0.33; *p* = 0.06, Wilcoxon signed-rank). The two Off-sustained cells did not show consistent changes in upstream or downstream noise parameters, but multiplicative noise increased strongly in both cells at high light (ratio low-to-high 0.23 and 0.03; data not shown).

## Discussion

A large body of work directly investigates the variability inherent in neural systems to inform our understanding of circuit function (Churchland et al., 2011; Goris et al., 2014; Gordus et al., 2015; Lin et al., 2015; Metzen et al., 2015; Zylberberg et al., 2016). Motivated by this, we have developed a new model that provides an improved representation of variability in a neuron’s response by incorporating multiple stochastic elements, representing various locations and types of noise present in neural circuits. The new model reduces bias in estimating circuit nonlinearities compared with oft-used models and provides estimates of multiple sources of variability at different locations within a circuit. The model reveals that changes in ambient light level produce systematic differences in both retinal circuit nonlinearities and sources of noise.

### Comparison of inferred nonlinearities and noise with experimental observations

The changes we observe in nonlinearities across light levels are consistent with previous work, with increasing rectification at higher light levels (Grimes et al., 2014). Yet the nonlinearities that we infer with the multistage noise model are generally more strongly rectified than those reported elsewhere and more strongly rectified than those found when assuming Poisson noise. Without explicitly accounting for certain kinds of noise, nonlinearities estimated from model fitting will appear more linear than the actual nonlinearity operating in the circuit. For example, if there is noise present upstream of a nonlinearity, it is expected that this noise will smear out the observed nonlinearity at the level of the outputs (as in Fig. 4*B*). Previous work has generally not attempted to disentangle the confounding effects of noise on the estimated nonlinearity. By allowing noise to be attributed to multiple potential circuit locations, the multistage noise model separates the effects of noise and deterministic model elements. This results in more sharply rectified estimates of nonlinearities than previously reported. These estimated nonlinearities provide better constraints on the operation of underlying circuit mechanisms and may reflect the dominance of particular mechanisms that are expected to be strongly rectified.

Our results show that all three noise sources in the model are required to account for ganglion cell response variability. A great deal of work points to a variety of origins of the noise in the retinal circuitry. Noise arising within the photoreceptors, and even in particular elements of the phototransduction cascade, has been studied extensively (Baylor et al., 1980; Schneeweis and Schnapf, 2000; Ala-Laurila et al., 2011; Angueyra and Rieke, 2013; Field et al., 2019). Other work points to several pieces of the retinal circuitry, particularly the bipolar cell output synapses, as significant sources of noise (Freed, 2000; Dunn and Rieke, 2006; Borghuis et al., 2009; Freed and Liang, 2014).

The relative contributions of different noise sources in the retinal circuitry could change with ambient light level, and we indeed see that the strength of different noise sources in the model varies systematically with light level. The two light levels tested here engage different retinal circuits before convergence at the RGC, which may change the relative contributions of noise sources directly or by altering the location and degree of nonlinearities in the circuitry, thereby effectively changing the location of noise relative to the nonlinearity (Field and Rieke, 2002; Grimes et al., 2014). Although the sources of variability in our model do not directly correspond to elements of the retinal circuitry, the observation of greater multiplicative noise at lower light levels is consistent with the fact that rod-mediated signals must traverse an additional synapse. Multiplicative noise in our model, which is present at the output of the nonlinearity and has strength that scales with nonlinearity output, is most similar to noise expected from stochastic vesicle release at synapses. Synaptic noise, which results largely from randomness in vesicle release, is often taken to be multiplicative or follow Poisson statistics (Rao et al., 1994; Freed, 2000; Moreno-Bote, 2014). In both cases, the variance in output scales with the output strength. This scaling of variance with mean has been previously observed in both On and Off ganglion cells in mouse retina (Murphy and Rieke, 2008).

### Limitations and extensions

LNP models and Poisson GLMs have gained widespread use in part because of their simplicity to fit to data. The model presented here is considerably more complex, although each of these additional components proved necessary to capture the full distribution of neural responses. Model parameters must be found via optimization on a relatively complex likelihood function and are not guaranteed to be unique. However, we find in practice that different initial conditions typically converge to the same set of parameters.

The work presented here captures the responses of a neuron to only a single temporal feature of the stimulus, and a spatially uniform stimulus was chosen to eliminate the need for spatial selectivity in the model. Selectivity to multiple spatiotemporal stimulus features, as has been observed in several systems (Rust et al., 2005; Slee et al., 2005; Fairhall et al., 2006), could be incorporated with straightforward extensions of the model. For example, if pathways that result in selectivity to two temporal features are assumed to converge before the nonlinearity, selectivity to these features can be incorporated by simply providing a weighted combination of these two features as the input to the model, adding one additional model parameter. Selectivity to spatial features of the stimulus is also relatively straightforward to incorporate if one assumes that the receptive field is composed of multiple identical spatial subunits (i.e., each subunit is characterized by the same nonlinear function). This model would require additional parameters to characterize the relative weighting of each subunit. Subunit models of RGC responses typically require only four to six subunits to capture responses well, suggesting that this addition is likely to be computationally tractable (Turner and Rieke, 2016; Shah et al., 2019).

A useful extension of this model would operate at finer timescales, predicting individual spikes rather than spike counts within a window of time (∼100 ms). Predicting responses in longer windows reduced the need for incorporating response history dependence. Indeed, we found that incorporating dependence on the response in the previous time bin did not improve our model predictions. However, refractory and other spike history effects would be necessary to make predictions at finer timescales. History dependence has been shown to improve model accuracy in a number of contexts, including in the retina (Berry and Meister, 1997; Pillow et al., 2005; Jolivet et al., 2006; Mensi et al., 2012). In particular, including refractory effects can help account for sub-Poisson variability, which is observed under certain stimulus conditions (Berry and Meister, 1998). Some models have even included two stochastic elements along with history dependence, although simplifying assumptions about the shape of the nonlinearity and/or the time course of history dependence are generally made (Keat et al., 2001; Vidne et al., 2012). Although adding additional history dependence is a conceptually straightforward extension of the model, it would require several additional parameters to characterize dependence on spike history.

Ideally, the model ought to optimize parameters of stimulus and spike history filters at the same time as nonlinearity and noise parameters. In the present study, we found linear filters using standard reverse correlation methods. Simultaneous optimization of these filters would require the addition of multiple new parameters, which could substantially slow optimization. Careful parameterization of these filters, incorporation of statistical priors, or additional simplifying assumptions may be required for this approach to be computationally tractable.

The general framework presented here could be easily modified to make use of different distributions for each noise source. We presented two slightly different versions, which incorporated different but closely related distributions for downstream noise. One could similarly modify upstream or multiplicative noise distributions, as called for by different datasets. Parameter inference will to some extent depend on these choices in model selection. We find, however, for the two models presented here that inferred nonlinearities are generally robust to this distinction and that inferred noise parameters change in small but systematic ways.

In conclusion, the model presented here holds several advantages over models that include a single source of variability. First, it is able to more accurately recover the nonlinearity in circuits in which noise is not dominated by a single source. Second, it provides better predictions of overall variability and has the ability to attribute variability to different sources. Given the importance of noise in shaping the flow of information through a circuit, it is important that a model capture features of this variability in the neural responses. This work opens the door to two potentially fruitful lines of future work: (1) extending the model to include additional features of stimulus and history dependence, and (2) conducting additional experiments to more closely link the sources of variability in the model to features of the biological circuit.

## Synthesis

Reviewing Editor: Nicholas J. Priebe, University of Texas at Austin Decisions are customarily a result of the Reviewing Editor and the peer reviewers coming together and discussing their recommendations until a consensus is reached. When revisions are invited, a fact-based synthesis statement explaining their decision and outlining what is needed to prepare a revision will be listed below. The following reviewer(s) agreed to reveal their identity: NONE. Note: If this manuscript was transferred from JNeurosci and a decision was made to accept the manuscript without peer review, a brief statement to this effect will instead be what is listed below.

The authors have addressed all remaining issues.
